# Characterization of an *Uncinocarpus reesii*-expressed recombinant tube precipitin antigen of *Coccidioides posadasii* for serodiagnosis

**DOI:** 10.1371/journal.pone.0221228

**Published:** 2019-08-14

**Authors:** Jieh-Juen Yu, Eric Holbrook, Yu-Rou Liao, Robert Zarnowski, David R. Andes, L. Joseph Wheat, Joshua Malo, Chiung-Yu Hung

**Affiliations:** 1 South Texas Center for Emerging Infectious Disease and Department of Biology, University of Texas at San Antonio, San Antonio, Texas, United States of America; 2 MiraVista Diagnostics, Indianapolis, Indiana, United States of America; 3 Department of Medicine, School of Medicine & Public Health, University of Wisconsin-Madison, Madison, Wisconsin, United States of America; 4 Department of Medicine, University of Arizona College of Medicine, Tucson, Arizona, United States of America; Oklahoma State University, UNITED STATES

## Abstract

Early and accurate diagnosis of coccidioidomycosis, also known as Valley fever, is critical for appropriate disease treatment and management. Current serodiagnosis is based on the detection of patient serum antibodies that react with tube precipitin (TP) and complement fixation (CF) antigens of *Coccidioides*. IgM is the first class of antibodies produced by hosts in response to coccidioidal insults. The highly glycosylated β-glucosidase 2 (BGL2) is a major active component of the TP antigen that stimulates IgM antibody responses during early *Coccidioides* infection. The predominant IgM epitope on BGL2 is a unique 3-O-methyl-mannose moiety that is not produced by commonly used protein expression systems. We genetically engineered and expressed a recombinant BGL2 (rBGL2ur), derived from *Coccidioides*, in non-pathogenic *Uncinocarpus reesii*, a fungus phylogenetically related to the *Coccidioides* pathogen. The rBGL2ur protein was purified from the culture medium of transformed *U*. *reesii* by nickel affinity chromatography, and the presence of 3-O-methyl mannose was demonstrated by gas chromatography. Seroreactivity of the purified rBGL2ur protein was tested by enzyme-linked immunosorbent assays using sera from 90 patients with coccidioidomycosis and 134 control individuals. The sensitivity and specificity of the assay with rBGL2ur were 78.8% and 87.3%, respectively. These results were comparable to those obtained using a proprietary MiraVista Diagnostic (MVD) IgM (63.3% sensitivity; 96.3% specificity), but significantly better than the ID-TP assay using non-concentrated patient sera (33.3% sensitivity; 100% specificity). Expression of rBGL2ur in *U*. *reesii* retains its antigenicity for coccidioidomycosis serodiagnosis and greatly reduces biosafety concerns for antigen production, as *Coccidioides* spp. are biological safety level 3 agents.

## Introduction

Coccidioidomycosis is a fungal infection caused by *Coccidioides immitis* and *Coccidioides posadasii* in endemic areas including the southwest region of United States, Mexico, and South America. The reported incidence of coccidioidomycosis has greatly increased over the past 2 decades [1]. Humans and animals contract this mycosis primarily through inhalation of coccidioidal arthroconidia from the environment. Although the minimum number of spores needed to cause symptomatic disease in human is not known, intranasal inoculation with approximately 10 viable spores in BALB/c mice is sufficient to cause disseminated disease and death in two to three weeks post-challenge [2]. Because of their low infectious dose and capacity as airborne pathogens, *Coccidioides* species are listed as risk group 3 agents, and require biosafety level 3 (BSL3) containment.

Current diagnosis of this disease is often delayed because of a lack of prompt testing and a reliable testing method. Diagnosis of coccidioidomycosis relies on clinical presentation of the disease, radiographic findings, delayed type hypersensitivity (skin) test results, culture and histopathological examination of tissue biopsy, and serodiagnosis [3–7]. The spectrum of illness following *Coccidioides* infection ranges from a mild flu-like syndrome or an uncomplicated pneumonia to progressive pulmonary destruction or life-threatening, disseminated diseases, which may involve skin, bone, muscle, and/or the central nervous system [3]. The manifestation of most early coccidioidal infections resembles other respiratory infections caused by bacteria and viruses. Some reports have shown that coccidioidomycosis is responsible for 15%-29% of patients presenting with community acquired pneumonia (CAP) in endemic regions, whereas less than 5% of CAP patients are tested for coccidioidomycosis [8, 9]. Culture and histological examination of patient sputum and tissue biopsy samples to detect the presence of *Coccidioides* often give false negative results [10]. Delayed type hypersensitivity tests cannot distinguish pre-exposed individuals from patients with active coccidioidal disease [7]. Therefore, detection of specific antibody responses to *Coccidioides* antigens is usually required to establish diagnosis of coccidioidomycosis.

The application of enzyme-linked immunosorbent assays (ELISAs) to measure specific IgM and IgG responses to tube precipitin (TP) and complement fixation (CF) antigens has increased in popularity because it is rapidly performed and does not require referral to a reference laboratory, as do complement fixation and immunodiffusion tests. Coccidioidal TP antigen is a complex containing at least 2 glycosylated proteins [11]. The major TP antigen is identified to be a heat-stable, 120 kDa β-glucosidase (BGL2) protein [12–14]. The predominant IgM-reactive epitope is mapped to a 3-O-methyl-mannose moiety that is an atypical carbohydrate. This unique sugar has only been identified in human pathogens such as *Mycobacterium* and *Coccidioides* species and a non-pathogenic fungus, *Uncinocarpus reesii* [12, 15, 16]. Crude coccidioidal extracts containing the native TP antigen have been widely used in clinical diagnosis kits. Although *Coccidioides* culture extract is a specific antigen source, this antigen preparation is labor-intensive and requires BSL3 laboratory confinement. One solution is to generate recombinant antigens that retain their antigenicity for coccidioidomycosis serodiagnosis. In this study, we genetically engineered and expressed a recombinant *Coccidioides posadasii*-derived BGL2 (rBGL2ur) in *U*. *reesii* and assessed immunogenicity of the purified protein using sera obtained from coccidioidomycosis patients

## Materials and methods

### Construction of pCE-TP plasmid

**Fig 1:**
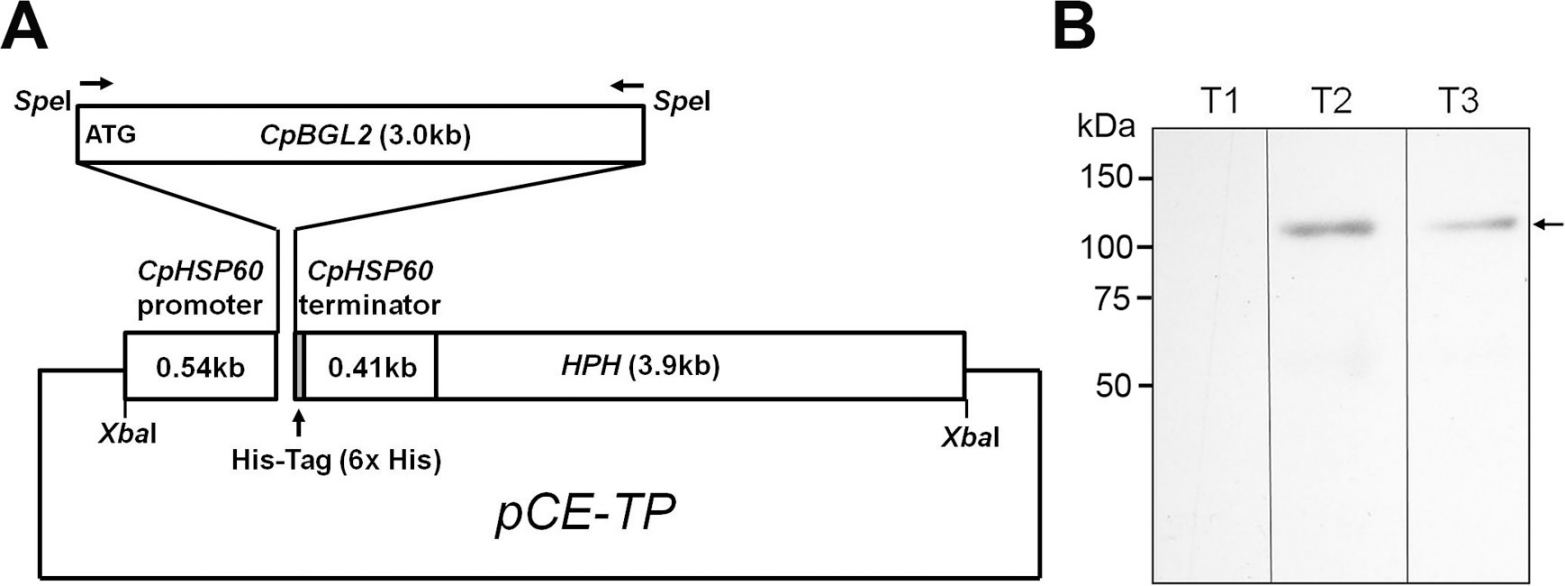
Generation of *Uncinocarpus reesii* transformants expressing recombinant BGL2. (A) An illustration of the pCE-TP plasmid used for transformation. Primer pairs (arrows) containing *SpeI* restriction sites were used to amplify the *Coccidioides posadasii BGL2* gene. The newly created *BGL2* gene was flanked with an *XbaI* site at each end in the *pCE-TP* plasmid. *XbaI* was used to release the BGL2 expression cassette prior to transforming *U*. *reesii*. (B) Total protein extracts of putative pCE-TP transformants T1-T3 were separated by SDS-PAGE and transferred onto PVDF membranes for western blot analysis. Expression of histidine-tagged rBGL2ur (arrow) was detected by probing with anti-histidine-tag antibody.

The pCE plasmid containing the promoter and terminator of the *C*. *posadasii* heat shock protein gene (*CpHSP60;* GenBank Accession No. U81786) and a histidine-tag sequence in front of the terminator was constructed in the pAN7-1 vector backbone (GenBank Accession No. Z32698) with a hygromycin resistance gene (*HPH*) for antibiotic selection [17]. We engineered a *SpeI* site and 2 *XbaI* sites on the pCE plasmid as illustrated in Fig 1A for easy cloning. The full-length *BGL2* gene (GenBank Accession No. AF022893.1) was PCR amplified using genomic DNA samples obtained from *Coccidioides posadasii* isolate C735 and one pair of *BGL2* gene-specific primers with an engineered *SpeI* restriction site at each end of the DNA fragment for subsequent cloning (5’-CCACTAGTATCTCACAATGTGG and 5’-TTACTAGTCGAAGACGGGGCTAG). The *SpeI*-digest of the *BGL2* gene amplicon (~3 kilobases) was cloned into the *SpeI*-digested pCE to form pCE-TP using standard molecular cloning methods. This plasmid was maintained and propagated in transformed *Escherichia coli* strain, TAM-1 for subsequent *U*. *reesii* transformation.

### Fungal transformation

*Uncinocarpus reesii* UAMH 3881 (ATCC 34534; American Type Culture Collection, Manassas, VA, USA) was grown on GYE agar (1% glucose, 0.5% yeast extract, 1.5% agar) at 30°C for 3 weeks to produce arthroconidia for transformation. Transformation of *U*. *reesii* was performed using a method that has been employed successfully for *Coccidioides spp*. [18, 19]. Briefly, germ tubes from *U*. *reesii* arthroconidia were digested with lysing enzymes (Sigma-Aldrich, St. Louis, MO, USA; L1412), Driselase (Sigma-Aldrich, D8037) and recombinant coccidioidal chitinase 1 [17] to produce protoplasts [18]. The pCE-TP plasmid was linearized by *XbaI* digestion and then incubated with *U*. *reesii* protoplasts in the presence of polyethylene glycol (Mn 3350, Sigma-Aldrich) and calcium ions to facilitate DNA uptake. Transformants were initially selected on GYE agar supplemented with 75 μg/ml hygromycin B followed by 3 subsequent passages on GYE agar plates containing 100 μg/ml hygromycin B to obtain stable pCE-TP transformed clones. Hygromycin at a lower concentration (75 μg/ml) was initially used to select transformants that were heterokaryotic hyphal cells. Subsequently a higher concentration (100 μg/ml) was applied to obtain homokaryotic transformants. Confirmation of the pCE-TP-transformed strains of *U*. *reesii* was conducted using a PCR screening method. Genomic DNA samples were prepared from fungal mats (~1 cm^2^) of each stable transformant for PCR screening, as previously reported [17] to identify *U*. *reesii* clones harboring the exogenous *Coccidioides BGL2* gene (3.0 kb) using the two *BGL2* cloning primers described above.

### Western blot analysis of rBGL2ur expression

Expression of histidine (His)-tagged protein from the PCR *BGL2*-positive *U*. *reesii* clones was examined by western blot analysis using an anti-His-tag monoclonal antibody (Sigma-Aldrich). The fungal strains were grown in 2 ml GYE along with 75 μg/ml hygromycin B for 5 days at 30°C, followed by 24 h of growth at 37°C. Total crude protein extracts were prepared from 0.3 ml of culture (hyphae plus media) by sonication. An aliquot of 15 μl from each sample was separated by sodium dodecyl sulfate-polyacrylamide gel electrophoresis (SDS-PAGE), transferred onto polyvinylidene difluoride membranes (Bio-Rad Laboratories, Inc. Hercules, CAL, USA), and probed with an anti-His-tag monoclonal antibody.

### Expression and purification of rBGL2ur

Seed culture of the *U*. *reesii* TP antigen expressing strain (URtp) was grown in 20 ml liquid GYE medium containing 75 μg/ml hygromycin on a rotary shaker at 30°C. The overnight seed culture was then used to inoculate multiple bottles of 350 ml GYE medium (at a 1:100 ratio) in a 1 l Erlenmeyer flask with a cotton stopper. The cultures were incubated in a shaker for 5 days at 30°C followed by heat shock at 37°C for two days to induce rBGL2ur expression. Expressed rBGL2ur was purified from the URtp culture media. Culture filtrates were collected by passing fungal culture through Whatman Grade 1 filter paper in a filtration unit that was attached to a vacuum system. Salting out of proteins in culture filtrates was achieved by adding ammonium sulfate (NH_4_)_2_SO_4_ to 90% saturation on ice. The protein precipitate was pelleted and then solubilized in binding buffer containing 50 mM Tris-HCl, 0.5 M NaCl and 2 M urea, pH 7.5. Nickel affinity chromatography was conducted using HisPur Ni-NTA Resin under denaturing conditions with 2 M urea following the manufacturer’s protocol (Thermo Fisher Scientific, Waltham, MA, USA). The bound protein was eluted using binding buffer supplemented with 200 mM imidazole. The eluted protein was dialyzed against Tris-HCl saline buffer (TBS) and concentrated by filtration centrifugation using Amicon Centrifugal Filter Units (10 kDa molecular weight cut-off, Millipore, Bedford, MA, USA).

### 3-O-methyl-mannose determination

Isolated rBGL2ur was subjected to carbohydrate profiling using gas chromatography (GC) analysis to determinate whether rBGL2ur possesses the TP antigen determinant sugar moiety, 3-O-methyl-mannose. Monosaccharides were detected and quantified by gas liquid chromatography-flame ionization detector (GLC-FID) on a Shimadzu GC-2010 system after conversion to alditol acetate derivatives, as previously described [20]. A Crossbond 50% cyanopropylmethyl/50% phenylmethyl polysiloxane column was used (15 m × 0.25 mm with 0.25 μm film thickness, RTX-225, Restek, Bellefonte, PA, USA). The GLC conditions were as follows: injector at 220°C, detector at 240°C, and a temperature program of 215°C for 2 min, then 4°C/min up to 230°C before holding for 11.25 min, run at constant linear velocity of 33.4 cm/sec and split ratio of 50:1. Pure 3-O-methyl-mannose (Omicron Biochemicals, South Bend, IN, USA) was used as a reference.

### Clinical specimens

A total of 90 serum samples from individuals with coccidioidomycosis were included, and 134 control samples from healthy individuals in endemic (n = 100) or non-endemic (n = 34) regions. Coccidioidomycosis cases were determined by a positive result for at least one of the following diagnostic methods, including *Coccidioides* culture, direct spherule visualization by histopathology or cytopathology, and any commercially available serological methods (ELISA, CF, immunodiffusion) [5]. Chart reviews were performed by experienced clinicians from endemic regions to verify the diagnosis of coccidioidomycosis [5]. Controls also included “clinical controls” (CC) [n = 17], which represented individuals with non-coccidioidomycosis diagnosis, as determined by chart review [5].

Residual patient samples were sent to MiraVista Diagnostics (MVD) from the University of Arizona. Individuals provided informed consent and patient information was handled in accordance with University of Arizona IRBs [5]. Samples provided to MVD were deidentified in accordance with IRB 16-MVIS-102 (Pearl IRB Indianapolis, IN). For testing, all healthy and coccidioidomycosis cases were randomized and blinded to the individual performing the experiments. Normal healthy serum was purchased from blood banks as described previously [5].

### Enzyme-linked immunosorbent assay

The antigenicity of rBGL2ur was assessed using ELISA. Sensitivity and specificity were assessed using 224 serum samples, as described above. All samples were blinded and screened using a variation of the previously described MVD ELISA method [5]. Plates were coated with 100 ng per well of rBGL2ur and blocked with StartingBlock (Thermo Scientific). Serum samples were diluted 1:250 in blocking solution before being added to each well. Anti-*Coccidioides* antibodies were then detected by the addition of horse radish peroxidase-labeled anti-human IgM (Thermo Scientific). Receiver operating characteristic (ROC) curves were calculated using coccidioidomycosis cases and controls using MedCalc statistical software for windows version 12.3.0 (MedCalc Software, Ostend, Belgium). Results of *Coccidioides* ID-TP and ID-CF tests were obtained by retrospective chart review. Additional coccidioidomycosis diagnoses using MVD IgG and IgM ELISA with a proprietary MVD *Coccidioides* antigen were performed as in our previous report [5].

Independent evaluation of serological reactivity of the purified rBGL2ur was also conducted at The University of Texas at San Antonio using a bank of *Coccidioides* reference sera (n = 10) obtained from the MRL Reference laboratory in 1996 and 10 control sera purchased from Innovative Research Inc. (Michigan, IL, USA). Patient information was anonymized and de-identified. The purified rBGL2ur was coated onto a 96-well microplate at a concentration of 100 ng/50 μl PBS per well overnight at 4°C. Each well was blocked with 100 μl of 1% bovine serum albumin (BSA) in PBS for 2 h and then washed with PBS containing 0.05% Tween 20 (PBST) once. The wells were then incubated with 100 μl human serum samples (1:50 dilution in PBS/0.1% BSA) for 1 h, followed by an incubation with 100 μl of secondary antibody [goat anti-human Ig(H+L)] conjugated with alkaline phosphatase (KPL, 1:2,000 dilution) for 1 h. After a final washing step, bound antibody was detected in the wells by addition of 100 μl of alkaline phosphatase substrate and absorbance was measured spectroscopically at a wavelength of 595 nm.

## Results

### Generation of genetically transformed *Uncinocarpus reesii* producing *Coccidioides* BGL2 protein

The pCE-TP plasmid illustrated in Fig 1A was constructed so as to contain the *C*. *posadasii BGL2* gene under the control of the *CpHSP60* promoter and terminator. *U*. *reesii* transcription and translation systems are able to express the gene downstream of the *CpHSP60* promoter, as we previously reported [17]. A His(6x)-tag nucleotide sequence was introduced at the 3’ end of the *BGL2* open reading frame followed by a stop codon. Additionally, the pCE-TP plasmid contained a hygromycin-B-phosphotransferase encoding gene (*HPH*) flanked by a promoter element of the *gpdA* gene and a terminator region of the *trpC* gene of *Aspergillus nidulans*. This *HPH* gene cassette is commonly used for creating *Coccidioides* transformants that are resistant to hygromycin B at up to 100 μg/ml. The inserted DNA fragment of *BGL2* was sequenced to confirm that it was identical to the reported gene (GenBank Accession No. AF022893.1). *XbaI*-linearized pCE-TP was used to transform protoplasts of *U*. *reesii* and allow random integration of the *XbaI*-digested fragment into its genome. Positive clones were grown on GYE agar plates with hygromycin at an initial concentration of 75 μg/ml. Subsequently, the hygromycin concentration was increased to 100 μg/ml to enrich for homokaryotic transformants. Transformants were subjected to PCR screening using gene-specific primers to identify potential BGL2-expressing transformants. Three transformants were randomly selected for further assessment of rBGL2ur expression levels by western blot analysis with an anti-His-Tag antibody. The deduced molecular weight of rBGL2ur (864 amino acids) is 93.6 kDa, while the anti-His-Tag antibody interacted with a protein band at approximately the 120 kDa region in transformants T2 and T3 (Fig 1B). This result implied that the reacting protein was highly glycosylated. The results showed that the T2 transformant had the highest expression level compared to the other tested clones. The T2 clone was designated URtp and used for subsequent rBGL2ur isolation and characterization. The NCBI accession number of this engineered *BGL2* gene construct encoding rBGL2ur is MK693019.

**Fig 2:**
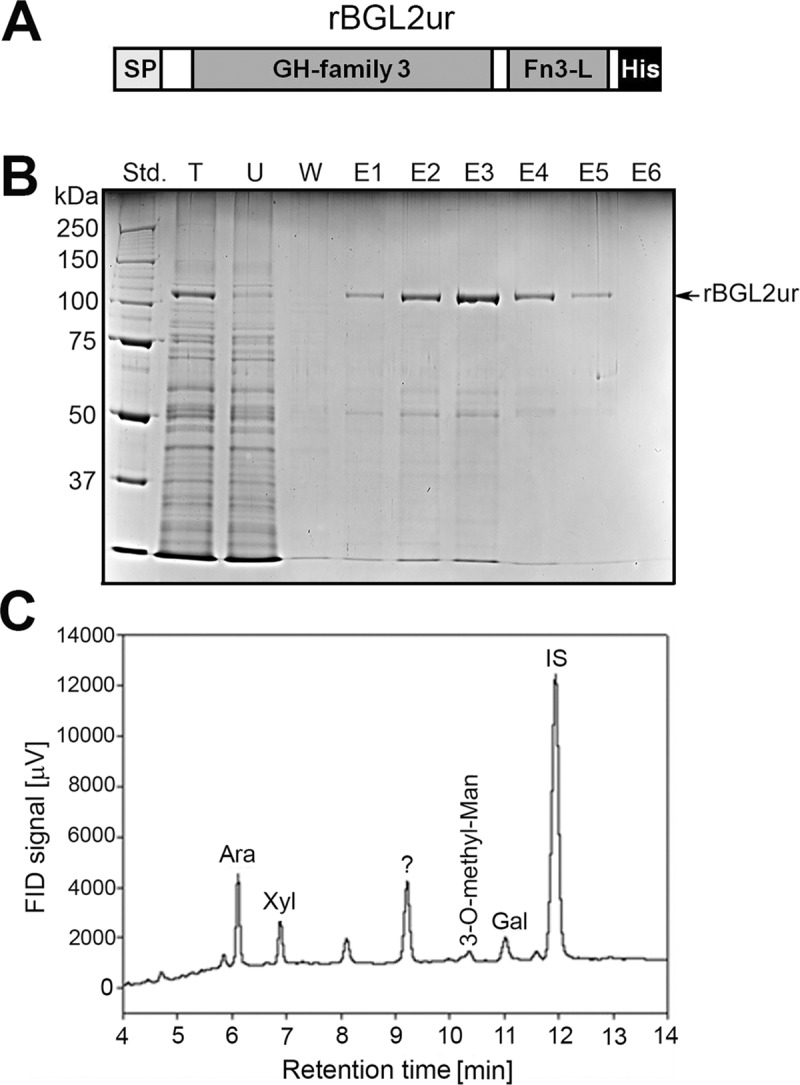
Isolation of rBGL2ur from pCE-TP transformed *U*. *reesii*. (A) An illustration of rBGL2ur protein domains depicting a cleavable N-terminus signal peptide (SP), a glycosyl hydrolase family 3 domain, a fibronectin type III-like domain, and a C-terminus His(6x)-tag. (B) pCE-TP transformant T2 (URtp) was cultured in GYE medium and heat-shocked to enhance the production of rBGL2ur. Protein samples were prepared from culture filtrates by (NH_4_)_2_SO_4_ precipitation and subjected to Ni affinity chromatography. Fractions of total (NH_4_)_2_SO_4_ precipitin (T), column flow-through (unbound proteins, U), weak Ni-binding proteins in washing buffer (W), and rBGL2ur proteins eluted from an Ni-column (E1-E6) were separated by 10% SDS-PAGE and stained with Coomassie Brilliant Blue. (C) Purified rBGL2 was subjected to chemical deglycosylation and the released sugars were detected and quantified by gas liquid chromatography-flame ionization detector (GLC-FID) on a Shimadzu GC-2010 system. Inositol was used as an internal standard (IS). The results revealed the presence of arabinose (Ara), xylose (Xyl), galactose (Gal) and 3-O-methyl-mannose. The peak labeled with a question mark (?) indicates an unidentified product formed during the chemical derivatization of monosaccharides.

The deduced rBGL2ur sequence was predicted using the SignalP-5.0 algorithm to contain a cleavable 18 amino acid signal peptide at the N-terminus that targets the protein to secretion pathways (Fig 2A). Thus, rBGL2ur was isolated from culture filtrates of URtp after 2 days of heat shock induction. The rBGL2ur protein was first salted out with (NH_4_)_2_SO_4_ followed by nickel affinity chromatography. rBGL2ur was purified to greater than 90% homogeneity, with a yield of approximately 3 mg of protein per liter of culture (Fig 2B). The purified rBGL2ur fraction was separated by SDS-PAGE and the 120 kDa band was excised, digested with trypsin, and subjected to liquid chromatography-tandem mass spectrometry (LC-MS/MS). Proteomic analysis of the obtained mass spectra identified 10 peptides that matched the deduced rBGL2ur amino acids (Table 1). Of the 10 peptides, 2 peptides (IMAAYYK and KVASASTVLLK) matched amino acid sequences of both rBGL2ur and *U*. *reesii* BGL1. These results raised a concern of Ur-BGL1 contamination in the purified rBGL2ur. The amino acid sequences of rBGL2ur and Ur-BGL1 shared 84% amino acid identity. No peptide in the trypsin-digested rBGL2ur sample matched with Ur-BGL1 alone, suggesting these 2 peptides were indeed derived from rBGL2ur.

**Table 1:** Molecular masses of peptides derived from trypsin digestion of purified rBGL2ur protein.

Detected BGL2 sequence	AA Position in rBGL2ur^a^	Molecular mass (Da)	
		*Predicted*	*Calculated*
EFVSQLTLTEK	49–59	1293.71	1293.68
GVDVQLGPAVGPLGR	136–159	1433.90	1433.80
LDDMAVR	330–336	818.42	818.40
IMAAYYK	337–343	858.46	858.43
DEFGYLHAGGQEGYGR	361–376	1754.80	1754.76
VNQMVNVR	377–384	958.52	958.50
HAVIAR	387–392	665.42	665.40
KVASASTVLLK	393–403	1115.72	1115.69
SPFTWAATSEDYGVSILK	596–613	1971.02	1970.96
APQIDFEEGIFIDYR	621–635	1811.91	1811.87

^a^The rBGL2ur sequence has been deposited in NCBI (MK683019)

The rBGL2ur protein contains 12 potential N-glycosylation sites that have an N-X-T/S motif (NetGlyc 1.0 server: http://www.cbs.dtu.dk/services/NetNGlyc/). Furthermore, rBGL2ur contains 61 serine and 52 threonine residues, among which, S_31_, S_35,_ S_357,_ T_619,_ and T_802_ are predicted to be potential O-glycosylation sites [21]. It is estimated that carbohydrate contributes approximately 22.5% of rBGL2ur molecular weight. Monosaccharide analysis revealed that the glycosylated moieties of rBGL2ur contained glucose (Glu), mannose (Man), arabinose (Ara), xylose (Xyl), and galactose (Glc). The presence of the TP immune determinant, 3-O-methyl-mannose, in the purified rBGL2ur was also confirmed (Fig 2C).

### Assessment of rBGL2ur as a serodiagnostic antigen for coccidioidomycosis

**Fig 3:**
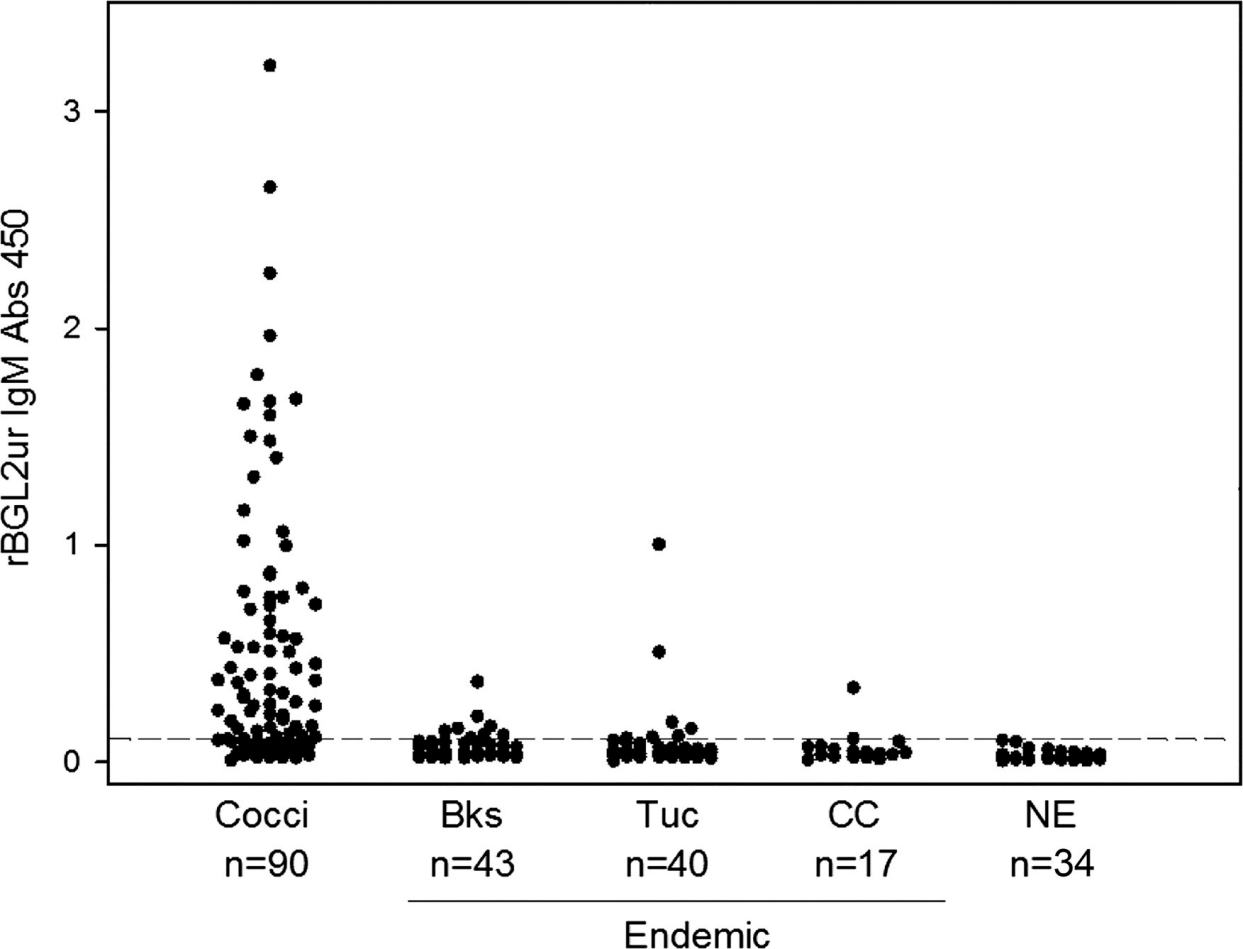
IgM antibody reactivity to purified rBGL2ur. IgM antibody levels were measured by optical absorbance readings at 450 nm (Abs 450) in samples from patients with coccidioidomycosis (Cocci), healthy individuals from Bakersfield, California (Bks), or Tucson, Arizona (Tuc), clinical controls (CC) from Tucson, Arizona and non-endemic (NE) healthy controls from Miami, Florida and Indianapolis, Indiana. The cutoff value of 0.096 Abs is indicated by the dashed line. The numbers under the labels are the total number of samples in each category.

We assessed the sensitivity and specificity of rBGL2ur as a serodiagnostic antigen for coccidioidomycosis in an MVD laboratory using 90 patient sera and 134 control samples obtained from Bakersfield, California, or Tucson, Arizona, clinical controls from Tucson, Arizona, as well as non-endemic healthy controls from Miami, Florida and Indianapolis, Indiana. A total of 43 patient sera were collected within 3 months upon initial disease diagnosis, while 47 sera were obtained after 3 months. They were also characterized by 63 and 27 cases of pulmonary and disseminated disease, respectively. Overall, the anti-rBGL2ur IgM-ELISA achieved 78.8% (71/90 patients) sensitivity and 87.3% specificity (false positives in 17 out of 134 control serum samples) based on a cut-off OD value of 0.096 as determined by ROC curves (Fig 3 and Table 2). This rBGL2ur-based ELISA had a trend of higher diagnostic sensitivity for cases of early (<3 months) or localized pulmonary infection than prolonged or disseminated infection (83.7% versus 74.5%). This might be due to fungal infection-induced early IgM antibody production against coccidioidal BGL2, in particular, its 3-O-methyl mannose sugar moiety, before isotype switching or weakening immune responses to BGL2. This is in contrast to the relatively delayed humoral responses to the CF antigen, as shown in Table 2 for the ID-CF assay, with higher positive detection rates for serum samples >3 months and those with disseminated disease. The rBGL2ur ELISA also had a much higher diagnostic sensitivity than classical ID-TP assays (78.8% versus 33.3%), although less specific (87.3% versus 100%). Compared to proprietary MVD *Coccidioides* antigen in IgM-ELISA, rBGL2ur is as good if not much better (Table 2: 78.8% versus 63.3% sensitivity; 87.3% versus 96.3% specificity) for coccidioidomycosis diagnosis.

**Table 2:** Comparison of sensitivities and specificities determined by ELISA and ID using different antigens.

Antigen/Test	Ab class		Sensitivity	Specificity	Time from Disease Diagnosis		Disease Manifestation	
			No. Patients (n = 90)	No. Controls (n = 134)	<3 months (n = 43)	>3 months (n = 47)	Pulmonary (n = 63)	Disseminated (n = 27)
rBGL2ur/ELISA	IgM	95% CI^a^	0.425–0.690	0.043–0.079	0.585–1.070	0.224–0.402	0.506–0.849	0.125–0.432
		Mean (OD)±SE	0.558 ±0.067	0.061±0.009	0.825±0.119	0.313±0.044	0.677±0.086	0.278±0.075
		No. Positive (%)^b^	71 (78.8)	117 (87.3)	36 (83.7)	35 (74.5)	54 (85.7)	17 (63.0)
MVD/ ELISA	IgG	95% CI	23.1–30.2	3.01–4.51	23.2–35.4	20.3–28.3	22.7–30.5	19.0–34.8
		Mean (EU)^c^±SE	26.7±1.78	3.76±0.38	29.3±3.01	24.2±1.98	26.6±1.97	26.9±3.83
		No. Positive (%)^b^	78 (86.7)	124 (92.5)	37 (86.0)	41 (87.2)	57 (90.5)	21 (77.8)
MVD/ ELISA	IgM	95% CI	15.3–22.9	4.28–5.39	17.6–29.9	10.4–19.2	16.5–26.0	8.03–20.0
		Mean (EU)^c^±SE	19.1±1.91	4.83±0.281	23.8±3.06	14.8±2.19	21.2±2.38	14.0±2.91
		No. Positive (%)^b^	57 (63.3)	129 (96.3)	30 (69.8)	27 (57.4)	43 (68.3)	14 (51.9)
ID-CF	IgG	No. Positive (%)^d^	52 (57.8)	127 (99.2)	23 (53.5)	29 (61.7)	32 (50.8)	20 (74.1)
ID-TP	IgM	No. Positive (%)^d^	30 (33.3)	128 (100)	17 (39.5)	13 (27.2)	23 (36.5)	7 (25.9)

^a^ 95% confidence interval (CI)

^b^Positive samples were considered when their Abs 450nm was greater than the assay cut-off value of 0.096.

^c^The cut-off for antibody detection is 10 Effective Units (EU)

^d^Positive cases were determined by visualizing a precipitation arc formed at a titer ≥ 1:2.

**Fig 4:**
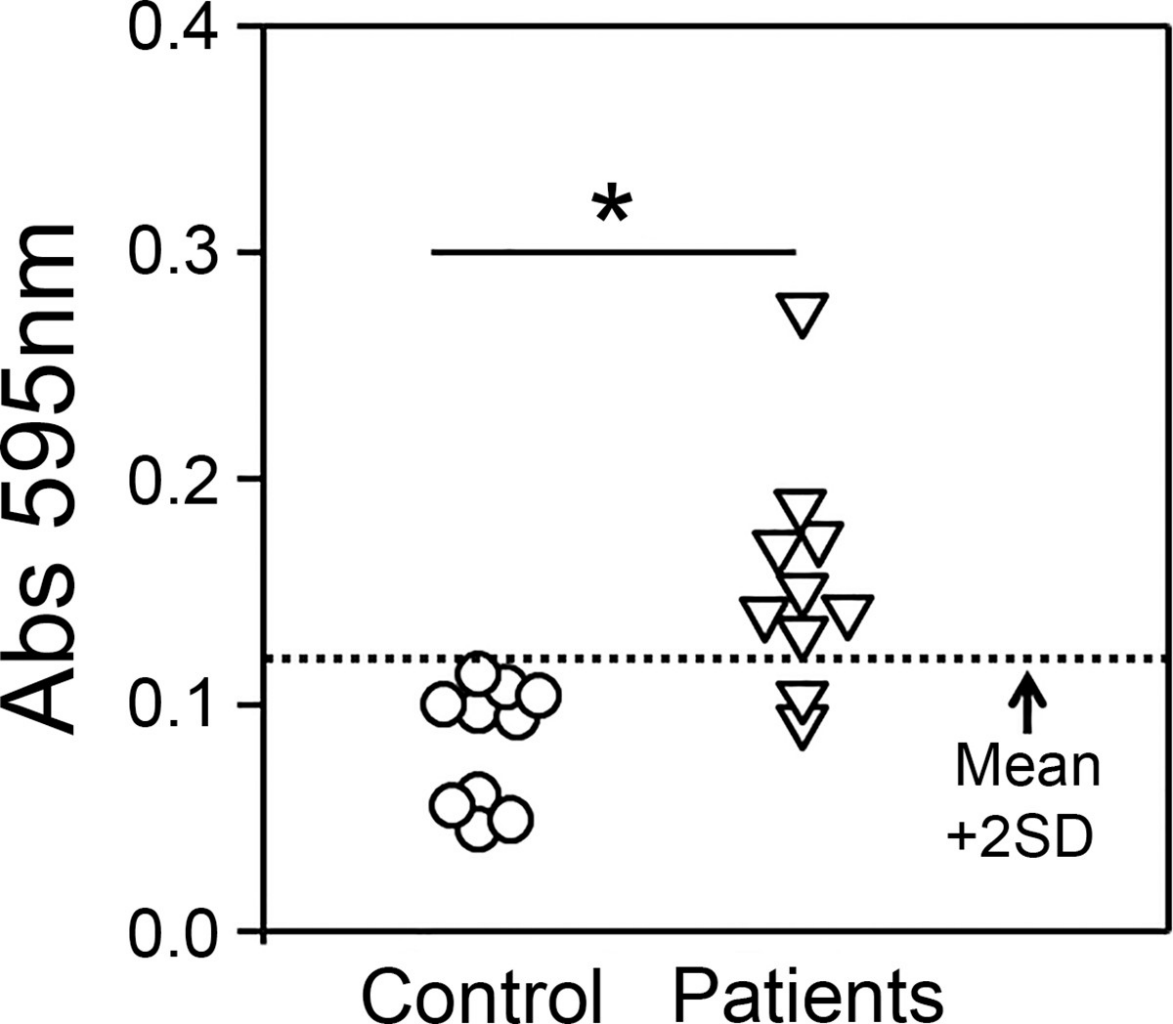
Consistency of rBGL2ur-ELISA for coccidioidomycosis diagnosis. A bank of coccidioidomycosis reference sera (n = 10) and control sera (n = 10) obtained from healthy donors who lived outside the endemic areas were diluted in PBS at 1:50 and incubated with a new batch of rBGL2ur in an indirect ELISA. An alkaline phosphatase-conjugated antibody reacting with human (H+L) chains of IgG, IgA, and IgM was used to detect seroreactivity with rBGL2ur (Student’s *t* test, *P = 00016).

We further assessed the consistency of rBGL2ur-ELISA as a diagnosis test at the University of Texas at San Antonio using independently obtained coccidioidomycosis reference sera (n = 10) and a different batch of rBGL2ur. Results of an indirect ELISA demonstrated that the different batch of rBGL2ur reacted with coccidioidomycosis patient antibodies significantly above the basal level of control sera (Fig 4). Specifically, 80% of patient sera had an optical density (OD_595_) reading equal or higher than the mean plus 2-fold standard deviation (Mean + 2SD) of the control group, while only one control sample was at this value. These data confirmed the specific antigenic reactivity of rBGL2ur with coccidioidal patient sera.

## Discussion

In this study, we generated a genetically altered *U*. *reesii* strain (URtg) that overexpresses rBGL2ur antigen of *Coccidioides posadasii*. We purified the rBGL2ur antigen by nickel affinity chromatography, confirmed its protein identity by amino acid sequence analysis and demonstrated the presence of a 3-O-methyl-mannose moiety in this recombinant protein. Furthermore, the antigenicity of rBGL2ur was confirmed using an ELISA to detect IgM in sera obtained from coccidioidomycosis patients and control individuals. The high detection sensitivity (78.8%) and specificity (87.3%) make the rBGL2ur antigen a useful component for clinical coccidioidomycosis diagnosis using an ELISA assay.

*Coccidioides* species are etiological agents designated as Biosafety Level 3 in *Biosafety in Microbiological and Biomedical Laboratories*, 5^th^ Ed. (https://www.cdc.gov/labs/BMBL.html). Accidental laboratory exposure to *Coccidioides* species is the major cause of laboratory-acquired fungal infections [22, 23]. Comparative genome analysis indicated that the nonpathogenic *U*. *reesii* is closely related to *Coccidioides* species [24]. ConA-bound fractions of mycelial preparations from *U*. *reesii* have been shown to react positively with a reference antibody in a coccidioidal ID-TP assay, making the fungus an ideal host to express recombinant coccidioidal BGL2 for serodiagnosis [16].

Glycosylation profoundly affects biological activity and antigenicity. *U*. *reesii* is the first reported expression system that can produce a glycosylated protein with 3-O-methy mannose moieties. Previously, we reported the expression of *Coccidioides* chitinase 1 (rCTS1ur) in *U*. *reesii* and demonstrated the CF antigenicity of the purified rCTS1ur [17]. We have now generated a second coccidioidal antigen in *U*. *reesii* with 3-O-methyl mannose glycosylation, further confirming the value of this non-pathogenic fungal expression system in the production of coccidioidal antigens. We postulated the *Coccidioides BGL2* gene in the pCE-TP construct was integrated into the *U*. *reesii* genome to maintain a stable hygromycin-resistant phenotype since the plasmid does not contain artificial chromosome elements such as autonomously replicating sequence and telomeres [25]. Function of the heat shock inducible CpHSP60 promoter was demonstrated in our previous report of rCTS1ur expression in pCE-CTS1 transformed *U*. *reesii* [17]. Thus, URtp is a genetically stable fungal transformant for overexpressing rBGL2ur following heat shock induction. In summary, although not fully-tested for clinical coccidioidomycosis diagnosis, generation of TP antigens using this reported rBGL2ur expression system is likely to have potential advantages over the production of crude antigen complexes from coccidioidal cultures in (1) enhancement of antigen purity and better antigen specificity, (2) increase of batch-to-batch consistency, (3) reduction of safety concerns and only BSL1 containment requirement, and (4) reduction of production costs.

*Coccidioides* BGL2 protein has been reported to be a cell wall-associated, as well as secreted, glycosyl hydrolase family 3 enzyme [11, 26, 27]. BGL2 protein is also a component of the TP-Ag complex [13, 14, 26, 27]. Although multiple antigenic determinants might be present in the TP-Ag complex to interact with patient IgM, a unique 3-O-methyl-mannose moiety is currently the only identified IgM epitope involving this antigen [12, 28, 29]. Detection of anti-TP IgM using an immunodiffusion assay takes 3–7 days to complete, and although it exhibits 100% specificity, it suffers from low sensitivity (~33%) when serum samples are not pre-concentrated. The development of an ELISA or lateral flow immunoassay using the rBGL2ur antigen may improve its sensitivity and shorten the required testing time, particularly for detecting this mycosis at the early stage of infection [5]. Indeed, utilization of rBGL2ur as an antigen in ELISA to detect patient IgM (Table 2, Fig 3 and Fig 4) has shown high diagnostic sensitivity and specificity.

Confirmatory diagnosis of coccidioidomycosis currently depends on positive serological test results for CF antigen using immunodiffusion or ELISA. The reported sensitivity of ID-CF and CF-ELISA ranges from 50–80%. The high false negative rate may contribute to delayed diagnosis. Navalkar *et al*. applied an immunosignature technology using a 96-peptide microarray to improve sensitivity and specificity of coccidioidomycosis diagnosis [30], but it is not clinically available to date. Serological tests for the presence of coccidioidal IgM may be also applied for early diagnosis of this mycosis [31, 32]. A positive ELISA result for IgM is believed to be associated with the initial antibody response seen in acute *Coccidioides* infections [33]. However, a recent study found that 82% of patients with an IgM-positive and IgG-negative ELISA result did not have coccidioidomycosis [34]. Whether the high false-positive rate in IgM ELISA was due to the usage of crude multi-component reference antigens in the diagnostic kit remains to be determined. Nonetheless, the reported findings and data from this study suggest that a combination of anti-rBGL2ur IgM-ELISA and anti-rCTS1ur IgG-ELISA could improve clinical serodiagnosis of potential coccidioidomycosis cases [35] and may potentially provide suitable cut-off values for assessment of coccidioidomycosis patients who may require immediate antifungal therapy [36].
